# Gefitinib (Iressa^®^) in metastatic patients with non-small cell lung cancer: preliminary experience in a Brazilian center

**DOI:** 10.1590/S1516-31802004000300010

**Published:** 2004-05-06

**Authors:** Auro del Giglio, Cristina Ito

**Keywords:** Lung neoplasms, Epidermal growth factor receptor, Administration & dosage Adverse effects, Prospective studies, Neoplasias pulmonares, Receptor do fator de crescimento epitelial, Administração & dosagem, Efeitos adversos, Estudos prospectivos

## Abstract

**CONTEXT:**

Patients with metastatic non-small cell lung cancer are deemed incurable, but they may derive some benefit from systemic palliative chemotherapy. Recently, with the introduction of epidermal growth factor receptor (EGFR) antagonists such as gefitinib (Iressa®), an effective and less toxic option is now available for the treatment of such patients**.**

**OBJECTIVE:**

To assess the activity and toxicity of gefitinib in a group of Brazilian patients.

**TYPE OF STUDY:**

Prospective, open label, non-randomized and non-controlled.

**SETTING:**

Clínica de Oncologia e Hematologia (CLIOH), São Paulo, Brazil.

**PATIENTS AND METHODS:**

From June 2002 to April 2003 we treated five patients with metastatic previously-treated non-small cell lung cancer (median of two previous chemotherapy regimens), using gefitinib at a dose of 250 mg orally on a daily basis, within a compassionate protocol sponsored by AstraZeneca. The patients’ median age was 65 years and two of them were male. Three had a performance status of 1, one of 2 and one of 3, on the ECOG (Eastern Cooperative Oncology Group) scale.

**RESULTS:**

We observed skin rash in two patients, diarrhea in three and arthralgia in two. One patient had a partial response and another had stabilization of her disease, as measured via imaging studies (which have lasted for more than 11 and 4 months, respectively), which were accompanied by significant decrease in tumor markers, whereas three patients worsened during treatment.

**DISCUSSION:**

New options of chemotherapy agents with favorable toxicity profiles are urgently needed for the treatment of metastatic non-small lung cancer patients who usually have short life expectancies. In our small series of five patients, we observed stabilization of the disease in two of them and the skin and gastrointestinal reactions often described in the literature in all of them. Two had arthralgia, not reported before.

**CONCLUSION:**

We concluded that gefitinib is an important addition to the therapeutic armamentarium for patients with metastatic non-small cell lung cancer.

## RESUMO

**CONTEXTO:**

Pacientes com carcinoma de pulmão não-pequenas células metastático são considerados incuráveis mas podem se beneficiar de quimioterapia sistêmica paliativa. Recentemente, com o advento de inibidores do receptor do fator de crescimento epitelial (EGFR), uma alternativa eficiente e menos tóxica surgiu para o tratamento destes pacientes.

**OBJETIVO:**

Testar em nosso meio a atividade e toxicidade do gefitinib.

**TIPO DE ESTUDO:**

Prospectivo, não aleatorizado e não controlado.

**LOCAL:**

Clínica de Oncologia e Hematologia (CLIOH), São Paulo, Brasil.

**PACIENTES E MÉTODOS:**

De junho de 2002 a abril de 2003, tratamos cinco pacientes com carcinoma de pulmão não-pequenas células metastático e previamente expostos a quimioterapia sistêmica (mediana do número de esquemas quimioterápicos prévios = 2) com gefitinib na dose de 250 mg ao dia, oral-mente, no contexto de um protocolo de disponibilização da droga patrocinado pela AstraZeneca. A idade média dos pacientes foi de 65 anos, dois eram de sexo masculino e três tinham um estado funcional de 1, um de 2 e um de 3 na escala do ECOG (Eastern Cooperative Oncology Group).

**RESULTADOS:**

Observamos erupção cutânea em dois, diarréia em três e artralgias em dois pacientes. Notamos uma resposta parcial e um paciente teve estabilização de sua doença com durações de 11+ e 4+ meses, respectivamente, ambas acompanhadas de significativa queda em seus marcadores tumorais. Três pacientes progrediram durante o tratamento.

**DISCUSSÃO:**

Novas opções de agentes quimioterápicos com perfis de toxicidade mais favoráveis são urgentemente necessárias para o tratamento de pacientes com câncer de pulmão não-pequenas células, que normalmente têm baixa expectativa de vida. Na nossa pequena série de cinco pacientes, observamos estabilização da doença em dois e reações de pele e gastrintestinais, freqüentemente descritas na literatura, em todos. Dois tiveram artralgia, não reportada até o momento.

**CONCLUSÕES:**

Concluímos que o gefitinib constitui importante adição ao armamentário terapêutico para tratamento de casos avançados de carcinoma de pulmão não-pequenas células.

## INTRODUCTION

Patients with non-small cell lung cancer are deemed incurable, but they may derive benefit from palliative chemotherapy. Unfortunately, however, life expectancy is low in such cases and may be hampered by chemo-therapy-related toxicity.

Gefitinib (Iressa→, ZD1839) is a tyro-sine kinase inhibitor that targets the intracellular domain of the epidermal growth factor receptor (EGFR), thereby blocking signal transduction pathways that are implicated in the proliferation and survival of cancer cells.^1^ Phase 1 trials on gefitinib defined a range of doses from 250 to 500 mg to be used in phase II studies.^2^ The toxicity observed in Phase I studies was mild and consisted mainly of skin rash and diarrhea.^2^ Phase II studies on patients with advanced previously-treated non-small cell lung cancer showed similar results for both 250 and 500 mg daily dosages.^3^ In these patients an objective response rate of between 10 and 20% has consistently been achieved and around an additional 40% of patients have derived clinical benefit due to disease stabilization. Furthermore, this clinical benefit has translated into improved quality of life for these patients.^4^

Although gefitinib has recently been approved by the Food and Drug Administration in the United States, in Brazil it was only available through a compassionate protocol sponsored by AstraZeneca, in which we included five patients. We report here our preliminary results with these patients.

## PATIENTS AND METHODS

This was a prospective, open label, non-randomized, non-controlled study that took place within a compassionate protocol sponsored by AstraZeneca. Patients were required to sign informed consent forms prior to their enrollment, as provided for as part of the protocol from this company. This compassionate protocol allowed the inclusion of patients with histologically proven non-small cell lung cancer in stages III or IV who had already received at least one previous chemotherapy regimen or were judged by the treating physician not to be good candidates for chemotherapy. Patients were excluded if they were receiving other chemotherapy or radiotherapy concomitantly, had other active malignant tumors, were not yet completely recovered from previous surgery, or were pregnant.

The therapy consisted of gefitinib provided by AstraZeneca, at a dose of 250 mg orally on a daily basis, until disease progression or significant toxicity precluded the continuation of the patient in the study, as judged by the treating physician. Patients were seen on a monthly basis and the disease response was assessed via imaging studies every two to three months. The toxicity was graded according to the World Health Organization (WHO) criteria.

## RESULTS

We included five patients with metastatic previously-treated non-small cell lung cancer (Table 1). All of them had adenocarcinoma. Their median age was 65 years and two of the reaction had not previously been reported in relation to gefitinib.

**Table 1:** Clinical characteristics of the patients included in a trial with gefitinib: all had metastatic non-small cell lung cancer

Patient initials Age		Sex	Histological type	Previous treatments	PS	Duration of treatment	Tumor response
VA	70	M	Epidermoid carcinoma	gemcitabine + cisplatin; docetaxel	2	3 months	Progression
LNM	66	M	Poorly differentiated carcinoma (via CK7 + surfactant + immunoperoxidase)	gemcitabine; vinorelbine; carboplatin	1	3 months	Progression
AOB	54	F	Epidermoid carcinoma	gemcitabine; vinorelbine; carboplatin + paclitaxel	1	4+ months	Stable disease
CHS	65	F	Adenocarcinoma	gemcitabine	1	11+ months	Partial response
LMR	55	F	Adenocarcinoma	vinorelbine	3	<1 month	Progression

*M: male; F: female; PS: Eastern Cooperative Group Performance Status Scale; CK7: cytokeratin 7.*

In our small series of five patients, we were also able to observe two patients with prolonged stabilization of their disease that is still ongoing and has so far lasted for 11 months in one case. Since all our patients were previously treated for metastatic disease, we feel that the results obtained in this group with poor prognosis are very encouraging and warrant further study of this drug.

patients were male. Three had a performance status of 1, one of 2 and one of 3, on the ECOG (Eastern Cooperative Oncology Group) scale. They had received a median of two chemotherapy regimens prior to gefitinib administration.

**Figure 1:**
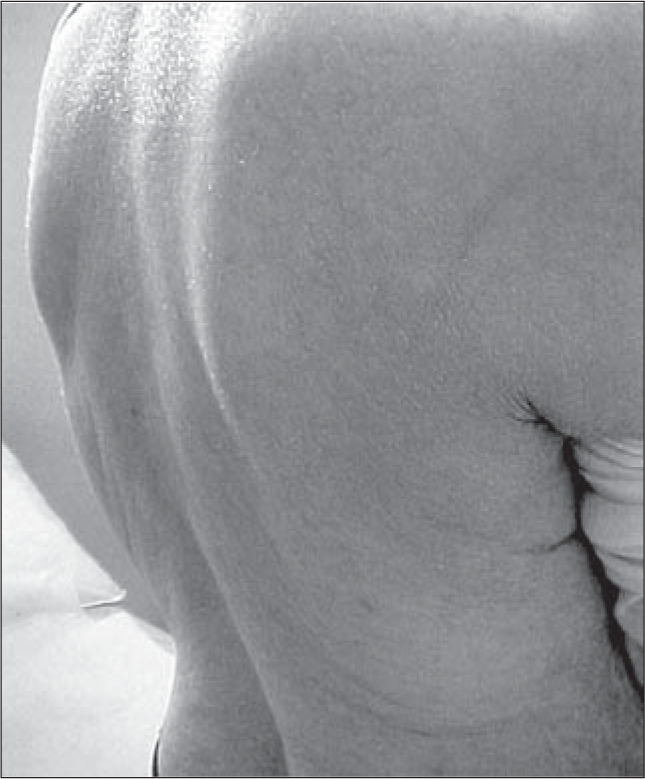
*Skin rash on the patient* CHS, *with adenocarcinoma of the lung, using gefitinib.*

We observed WHO level 2 skin rash in two patients (Figure 1). Three patients experienced diarrhea that was of WHO grade 1 in two of them and grade 4 in the third. This last patient with grade 4 diarrhea had been hospitalized before the gefitinib treatment because of severe diarrhea that resolved prior to enrollment in this protocol. At the time of enrollment, an extensive workup including gastrointestinal investigation was negative. Therefore, the relationship of his severe diarrhea with this drug is unclear.

One patient had to be admitted to hospital because of an episode of intestinal subocclusion that resolved spontaneously with conservative management. A small bowel series (SBS) showed only diffuse intestinal slowing with no anatomical obstruction. We could not ascertain whether this subocclusion was related to gefitinib because this patient is still receiving the medication without recurrence of this symptom. Two of our patients reported mild arthralgia.

**Figure 2:**
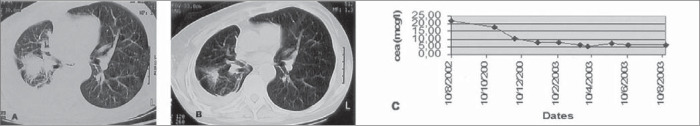
*Partial response seen in the patient* CHS, *with the CEA (carcinoembryonic antigen) values at different time points. Panel A: computed tomography scan of the chest in September 2002; Panel B: computed tomography scan of the chest in August 2003; Panel C: CEA levels throughout the treatment using gefitinib*.

One patient had a partial response that is still ongoing and has lasted for 11 months so far, and another has had stabilization of her disease for the last four months, as measured via imaging studies, without evidence of progression at the time of this writing. In these two cases, we also observed a significant decrease in serum tumor markers (Figure 2). Three of the five patients worsened during treatment and stopped using gefitinib.

## DISCUSSION

Patients with metastatic non-small cell lung cancer who have previously been exposed to chemotherapy have a poor prognosis with short life expectancy. Even though these patients may benefit modestly from second-line chemotherapy, its additional toxicity may not be warranted. Therefore, new options with a more favorable toxicity profile that can effectively control this disease are urgently needed. Gefitinib seems to fulfill this role for the treatment of such patients, since the phase I and II studies^2,3^ so far conducted show consistently that it can benefit about 50% of them and also improve their quality of life.^4^

In conformity with descriptions in the literature,^2,3^ we also observed skin and gastrointestinal toxic reactions in our patients. The most common ones were diarrhea and skin rash. Two of our patients complained of arthralgia: to our knowledge, this type of toxic reaction had not previously been reported in relation to gefitinib.

In our small series of five patients, we were also able to observe two patients with prolonged stabilization of their disease that is still ongoing and has so far lasted for 11 months in one case. Since all our patients were previously treated for metastatic disease, we feel that the results obtained in this group with poor prognosis are very encouraging and warrant further study of this drug.
